# Pan-American Notes

**Published:** 1901-07

**Authors:** 


### PAN-AMERICAN NOTES.

THE Exposition Hospital, which has been fully described in the JOURNAL, has had plenty of business since the grounds were opened. Over 1,600 patients have been treated, which indicates a large attendance at the fair. Visitors who may be taken ill are assured of the best of medical service and nursing.

FOUR new nurses were appointed by Supt. Adella Walters for the month of June at the Pan-American Hospital. The appointees are Miss Florence Hamilton and Miss Louise Greenwood of the Buffalo General Hospital; Miss Laura M. Jarvis of the Arnot Ogden Memorial Hospital, Elmira, and Miss Eleanor M. Alexander of the Kingston General Hospital, Toronto. Four others will be appointed for the next month.

AN X-RAY machine has been installed in Medical Director Park's office.

AMONG the conveniences added to the hospital recently is a Fox-Piper invalid bed. It was contributed by Harry Piper, a former alderman of Toronto. It appears to be admirably adapted for lifting and changing the position of heavy patients and for easing the pain of those suffering from fracture, paralysis or other similar helpless conditions.

THE infant incubators are attracting a great deal of attention. It is interesting to note the processes of development that can be witnessed here. Many a prematurely born child which would otherwise be lost is nurtured up to a vitality that makes it capable of independent life. As soon as a child is received it is given a bath in mustard-water, then two drops of brandy are placed in its mouth. Its body is next rubbed with alcohol, and the child is placed in the incubator, which is kept at a temperature of 96 degrees Fahrenheit for four or five days. It is removed regularly day and night every hour and a half to be fed about fifteen grams of nourishment with a nasal spoon.

A FILIPINO girl baby was born on the Midway June 18, 1901. This little girl is the first Filipino child born in this country. That she is lusty is evidenced by the power of her lungs, and she will soon begin to hold receptions. Her mother, of course, is very proud of the American Filipino, and was much concerned over the proper naming of the infant. All the village felt that this infant was an uncommon child and that a specially fitting name should be bestowed.

DR. CONEY of the baby incubators was called to the Indian Congress at 3.30 o'clock a.m. June 20th. An Indian baby had been prematurely born. The mother is Princess Ikishupaw, an Apache. The baby weighs only two pounds, two ounces. It is one of the smallest babies ever born. Dr. Coney had it removed to the baby incubators, where it was placed in one and where it can be seen. The father is Chief Many Tales, also an Apache.

THE United States Hospital Corps' camp, near the Government Building, is paved with broken stone, and is an exceedingly interesting exhibit. Dr. Edward L. Munson, captain and assistant surgeon U. S. Army, is in command and there are 100 men under him. The camp consists of 22 tents. The hospital wards are composed of twelve "A" tents, set up in the form of a Geneva cross. There is a tropical ward, a winter ward, a summer ward. If the rest of the United States Army was represented in proportion to the hospital corps there would be over 5,000 United States troops on the grounds. The Hospital Corps and equipment station at the exposition are such as is usually assigned to an army brigade which contains 5,380 men.

SOMEONE has estimated the cost of a visit to the exposition from New York. The figures are as follows:

| | |
|---|---|
| Fare from New York to Buffalo and return | $9.00 |
| Room for four nights | 3.00 |
| Meals | 4.10 |
| Admission to exposition | 1.50 |
| Admission to Midway shows | 4.00 |
| Guide book | .25 |
| Carfare to exposition | .30 |
| Seeing Buffalo | .30 |
| Fare to Niagara Falls | .50 |
| Niagara attractions | 1.10 |
| Incidentals | .95 |
| Total | $25.00 |

